# Machine Learning Models for Predicting Influential Factors of Early Outcomes in Acute Ischemic Stroke: Registry-Based Study

**DOI:** 10.2196/32508

**Published:** 2022-03-25

**Authors:** Po-Yuan Su, Yi-Chia Wei, Hao Luo, Chi-Hung Liu, Wen-Yi Huang, Kuan-Fu Chen, Ching-Po Lin, Hung-Yu Wei, Tsong-Hai Lee

**Affiliations:** 1 Department of Electrical Engineering National Taiwan University Taipei Taiwan; 2 Department of Neurology Chang Gung Memorial Hospital Keelung Taiwan; 3 Institute of Neuroscience National Yang Ming Chiao Tung University Taipei Taiwan; 4 Community Medicine Research Center Chang Gung Memorial Hospital Keelung Taiwan; 5 Department of Neurology Linkou Chang Gung Memorial Hospital Taoyuan City Taiwan; 6 College of Medicine Chang Gung University Taoyuan Taiwan; 7 Clinical Informatics and Medical Statistics Research Center Chung Gung University Taoyuan Taiwan; 8 Department of Emergency Chang Gung Memorial Hospital Keelung Taiwan

**Keywords:** cerebrovascular disease, acute ischemic stroke, machine learning, random forest, early outcome, prediction, explanation, SHapley Additive exPlanations

## Abstract

**Background:**

Timely and accurate outcome prediction plays a vital role in guiding clinical decisions on acute ischemic stroke. Early condition deterioration and severity after the acute stage are determinants for long-term outcomes. Therefore, predicting early outcomes is crucial in acute stroke management. However, interpreting the predictions and transforming them into clinically explainable concepts are as important as the predictions themselves.

**Objective:**

This work focused on machine learning model analysis in predicting the early outcomes of ischemic stroke and used model explanation skills in interpreting the results.

**Methods:**

Acute ischemic stroke patients registered on the Stroke Registry of the Chang Gung Healthcare System (SRICHS) in 2009 were enrolled for machine learning predictions of the two primary outcomes: modified Rankin Scale (mRS) at hospital discharge and in-hospital deterioration. We compared 4 machine learning models, namely support vector machine (SVM), random forest (RF), light gradient boosting machine (LGBM), and deep neural network (DNN), with the area under the curve (AUC) of the receiver operating characteristic curve. Further, 3 resampling methods, random under sampling (RUS), random over sampling, and the synthetic minority over-sampling technique, dealt with the imbalanced data. The models were explained based on the ranking of feature importance and the SHapley Additive exPlanations (SHAP).

**Results:**

RF performed well in both outcomes (discharge mRS: mean AUC 0.829, SD 0.018; in-hospital deterioration: mean AUC 0.710, SD 0.023 on original data and 0.728, SD 0.036 on resampled data with RUS for imbalanced data). In addition, DNN outperformed other models in predicting in-hospital deterioration on data without resampling (mean AUC 0.732, SD 0.064). In general, resampling contributed to the limited improvement of model performance in predicting in-hospital deterioration using imbalanced data. The features obtained from the National Institutes of Health Stroke Scale (NIHSS), white blood cell differential counts, and age were the key features for predicting discharge mRS. In contrast, the NIHSS total score, initial blood pressure, having diabetes mellitus, and features from hemograms were the most important features in predicting in-hospital deterioration. The SHAP summary described the impacts of the feature values on each outcome prediction.

**Conclusions:**

Machine learning models are feasible in predicting early stroke outcomes. An enriched feature bank could improve model performance. Initial neurological levels and age determined the activity independence at hospital discharge. In addition, physiological and laboratory surveillance aided in predicting in-hospital deterioration. The use of the SHAP explanatory method successfully transformed machine learning predictions into clinically meaningful results.

## Introduction

Cerebrovascular disease ranks as the second leading cause of death in the United States and the third cause of disability-adjusted life years (DALYs) globally in 2010 [1]. Ischemic stroke shows higher incidence and prevalence than hemorrhagic stroke. Ischemic stroke survivors commonly have disabilities and substantial function loss that significantly affect their quality of life. Outcome prediction provides a reference for doctors to select rehabilitation strategies and provides patients with decent expectations in the future [2,3]. Several studies have focused on stroke prediction by indicators collected at emergency room (ER) or first at ward admissions [4,5]. In the past, scores such as the Acute Stroke Registry and Analysis of Lausanne (ASTRAL), DRAGON, and SEDAN were used for stroke outcome prediction and proved more accurate than physicians [6]. Over the past few years, most research on stroke prediction has emphasized the use of machine learning, which achieves better performance in predicting stroke outcomes [7]. Recent studies on stroke prediction can be classified into three categories: studies investigating longitudinal data such as health insurance databases for predicting the probability of stroke occurrence, studies predicting recovery in a specific time using numerical data, and studies applying novel machine learning models such as computer vision models [8] or natural language processing models for more accurate diagnosis [9,10].

This work aimed to predict early outcomes using numerical data and applying novel machine learning models, including neural networks and gradient boosting machines for predictions. The specific goals were to predict the modified Rankin Scale (mRS) score at hospital discharge and deterioration during admission. We focused on model performance comparison, ranking of feature importance, and explanation of model predictions. We leveraged the SHapley Additive exPlanations (SHAP) to depict the stroke prediction models and guarantee that the models predict with a solid basis. For imbalanced prediction targets, preprocessing was performed with different resampling methods to balance the data set before model performance comparisons.

## Methods

### Database

Patient data were collected from January 1 to December 31, 2009, by the Stroke Registry in Chang Gung Healthcare System (SRICHS) [11]. SRICHS is a stroke registry system that prospectively collected patients’ clinical information with the ICD 9 diagnostic code 430-437 for acute ischemic and hemorrhagic stroke since 2007. The registry data were anonymized and deidentified before analysis. The data automatically downloaded from the hospital information system included demographic information, laboratory tests, examination reports, and structured information from the electronic medical chart. The data cleaning process included 2 steps. First, the data without the initial blood pressure recordings at admission, mRS at ward admission and discharge, and laboratory hemograms were removed. Second, the data with out-of-range scores on the National Institutes of Health Stroke Scale (NIHSS) were removed, which were attributed to misrecording. The Institutional Review Board of Chang Gung Memorial Hospital approved this study (no. 103-1519C, no. 201900732B0, and no. 201801763A3).

### Outcome Measurements

The primary target variable was the mRS at discharge [12]. To turn the prediction issue into a binary classification problem and compare our results directly with the existing methods, we discretized the mRS into two classes: good outcomes defined by mRS 0-2 and poor outcomes defined by mRS≥3.

The other primary outcome was in-hospital deterioration. The coding for deterioration included clinical condition worsening due to brain herniation, hemorrhagic transformation, neurological deterioration defined by an increase of 4 points or more in the NIHSS score compared to the admission score, and clinical deterioration due to medical problems. When there were specific causes for increases in the NIHSS scores by 4 points or more, such as brain herniation or hemorrhagic transformation, the patients were coded for these reasons; otherwise, we coded them for neurological deterioration. If mortality or critical conditions occurred owing to medical complications, we assigned them the code of in-hospital deterioration due to medical problems.

### Features in the Models

The following categories of features were included in the models: (1) demographic features: age, sex, smoking habit, alcohol consumption, height, weight, and BMI; (2) medical comorbidities: a history of previous stroke, ischemic heart disease, congestive heart failure, atrial fibrillation, diabetes mellitus (DM), hypertension, and hyperlipidemia; (3) stroke-related index: NIHSS total score and subscores at ER and ward admission and stroke onset-to-hospitalization interval; (4) initial physiological parameters at admission: initial systolic blood pressure (SBP) and diastolic blood pressure, heart rate, respiratory rate, and body temperature; (5) initial laboratory parameters of blood tests: hemogram including the white blood cell (WBC) count and its differential counts, red blood cell (RBC) count, hemoglobin, hematocrit and platelet counts, prothrombin time (PT), activated partial thromboplastin time, cholesterol and triglyceride profile, aspartate aminotransferase, alanine transaminase, blood urea nitrogen, creatinine, glucose, glycosylated hemoglobin, C-reactive protein, erythrocyte sedimentation rate, and homocysteine; (6) data of urine tests, including urine total protein and glucose levels.

### Data Visualization

Unsupervised clustering provided an explicit grouping of the data, and direct visualization of the clusters showed the natural distribution of data. The t-distributed stochastic neighbor embedding (t-SNE) is a nonlinear dimensionality reduction for visualization [13]. Let P be the joint probability distribution for high dimension, and Q for low dimension. The distance between the 2 similarity matrices could be expressed as: 
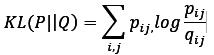
 A gradient descent was performed to minimize this score, and the gradient could be computed as: 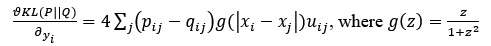


### Machine Learning Models

#### Support Vector Machine (SVM)

The SVM was used to construct a hyperplane to split the data into 2 classes and optimize the distance between all data points and the hyperplane [14]. For a set of {*x_i_*, *y_i_*}, *i* = 1, …, *N*, *x_i_* ∈ *Rdy_i_* ∈ {+1, –1}, the SVM found a vector *ω* such that *y_i_* (*ωTx_i_ – b*) > 0. The vector split the data into 2 classes. Many lines were available for splitting the set. The SVM optimized the solution by solving: 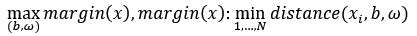 And retrieved the solution from: 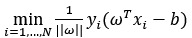


#### Random Forest (RF)

The RF algorithm was based on bagging and decision [15]. Bootstrap aggregating (bagging) used repeated random sampling and replaced the training set to create a subset, reduce variance, and improve accuracy. Each subset of the training set conducted a random selection with features. The aggregation combined all predictions and yielded the regression mean and classification mode.

#### Light Gradient Boosting Machine (LGBM)

LGBM is a gradient boosting framework using tree-based learning algorithms [16]. In LGBM, gradient-based one-side sampling (GOSS) and exclusive feature bundling (EFB) were the 2 main techniques to improve efficiency and scalability. GOSS kept those data with large gradients and randomly dropped those with small gradients and reduced the calculation cost. EFB bundled exclusive features to reduce feature dimensions. The feature bundles could improve training efficiency without losing accuracy.

#### Deep Neural Network (DNN)

The DNN model was trained with tuned parameters in neurons by adjusting their weights and bias values to make the model’s output closer to the ground truth [17]. If we set θ as all the parameters of the model and the input as *x* passing the neural network, F(θ), the output layer would generate the corresponding F(*x*, θ) = *y*ˆ. The embedding layer turned positive integers (indexes) into fixed-size vectors. The technique could avoid the sparse matrix obtained during the transformation of high-dimensional data into lower-dimensional data and turned categorical data into one-hot encoding data. In the DNN, the gradient descent algorithm solved the optimization problem by calculating the gradient of the loss function, updating the model's parameters in the opposite direction, and minimizing the loss. By selecting an optimal learning rate, a local minimum would be reached by iterations. Additional methods to optimize the model included batch normalization, which normalized the means and variances of each layer’s inputs [18]. Dropout avoided overfitting by randomly omitting a certain fraction of neurons on each training case [19].

### Data Processing

**Figure 1:**
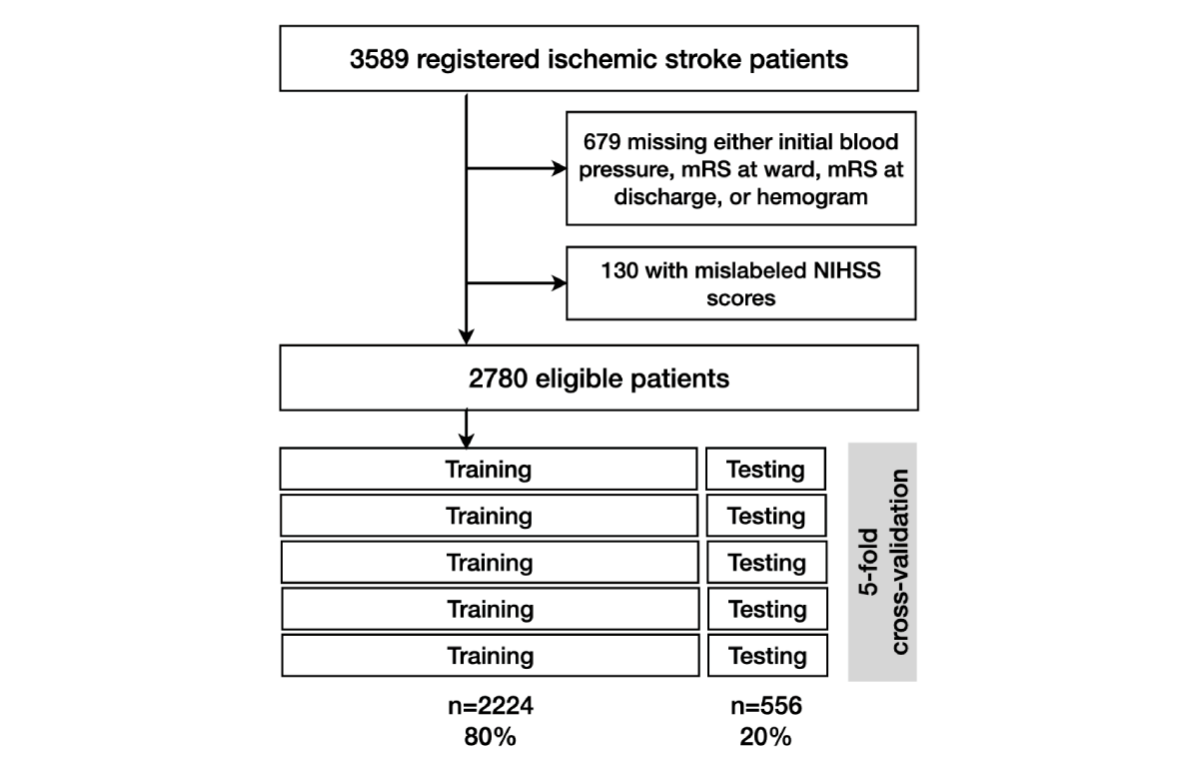
Data enrollment. After the initial enrollment of 3589 patients, data cleaning excluded 809 patients and left 2780 eligible patients. The enrolled data set underwent k-fold cross-validation. In 5 folds, the data set was randomly divided such that 80% was for training and 20% for testing in each fold. The results of cross-validation underwent performance comparison with the ground truth and are expressed as the area under the curve of the receiver operating characteristic curve. mRS: modified Rankin Scale; NIHSS: National Institutes of Health Stroke Scale.

We applied a min-max normalizer to the numerical data for data engineering, split the data into 5 folds, and performed 5-fold cross-validation for performance evaluation. Cross-validation is a suitable approach to estimate the performance of a model when the data set is small. During the process of 5-fold cross-validation, the data set was first divided into 5 groups; then, each group was used as an unseen testing set in turn, whereas the remainder of the data set served as the training set ( Figure 1). Notably, for DNN and LGBM, 10% of the training set was used as the validation set (tuning set) to prevent the overfitting problem and ensure that the model is trained well. Finally, the mean and SD of the testing accuracy in 5 rounds were evaluated as performance metrics.

Transformation of the multiclassification model to a binary model was performed to improve model performance. After training the RF and LGBM multiclassifiers, the output summed up the mRS outcome {0*,*1*,*2} as False, and mRS outcome {3*,*4*,*5*,*6} as True.

Between-model comparison was conducted to rate the SVM, RF, LGBM, and DNN. Model performance in terms of the prediction ability was evaluated using the average area under the curve (AUC) of the receiver operating characteristic (ROC) curve; clear interpretations of true positives and false positives were essential for the classification problem.

### Imbalanced Data

To handle imbalanced outcomes, 3 resampling methods were applied to make the 2 outcome classes more balanced. First, random under sampling (RUS) randomly dropped data from the majority class and often led to missing critical data. Second, random over sampling (ROS) randomly duplicated data of the minority class but sometimes led to overfitting of the minor samples. The third resampling method was the synthetic minority over-sampling technique (SMOTE) [20], which synthesized data from the minority class. The synthetic sample *x* is a point along the line segment joining *x_i_* and *x^i^*, where ***x***^0^*_i_* = ***x****_i_* + (***x***ˆ*_i_* − ***x****_i_*) × *δ* and the random number *δ* ∈ (0*,*1). The synthetic minority over-sampling technique-nominal continuous (SMOTE-NC) technique is the advanced modification of SMOTE and capable of handling mixed data sets of continuous and nominal features. The SMOTE-NC ran median computations for nominal features and nearest neighbor computations for mixed data. The algorithm gave those nominal features the value occurring in most k-nearest neighbors.

### Interpretation of Models

The SHAP, inspired by the Shapley value in game theory, assigned each feature a value of importance for a particular prediction [21]. The SHAP summary used kernel SHAP to estimate the Shapley value and visualized the prediction distribution among the feature values. For example, when approximating the original model *f* for a specific input *x*, local accuracy required the explanation model to match the output off for the simplified input *x′* that corresponded to the original input *x*: 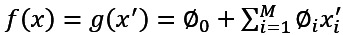


### Data Availability

Anonymized data not published within this article will be made available on request from any qualified investigator under the regulations of our institutional review board.

## Results

### Data Enrollment

Initial screening identified 3589 patients of admission due to acute ischemic stroke. The data cleaning steps excluded 679 patients for missing records of blood pressure, mRS, and hemograms. Another 130 patients were excluded for mislabeled NIHSS scores. The missing rate of all the features was under 10%. A total of 2780 eligible patients were enrolled. The data underwent 5-fold cross-validation. In each fold, the models randomly divided the whole data set into 80% data for training and 20% data for testing. The performance in each fold was compared with the ground truth and quantified in the AUC of ROC curves. The final AUC results were the means and SDs obtained from the 5-fold cross-validation (Figure 1).

### Prediction of mRS at Hospital Discharge

**Figure 2:**
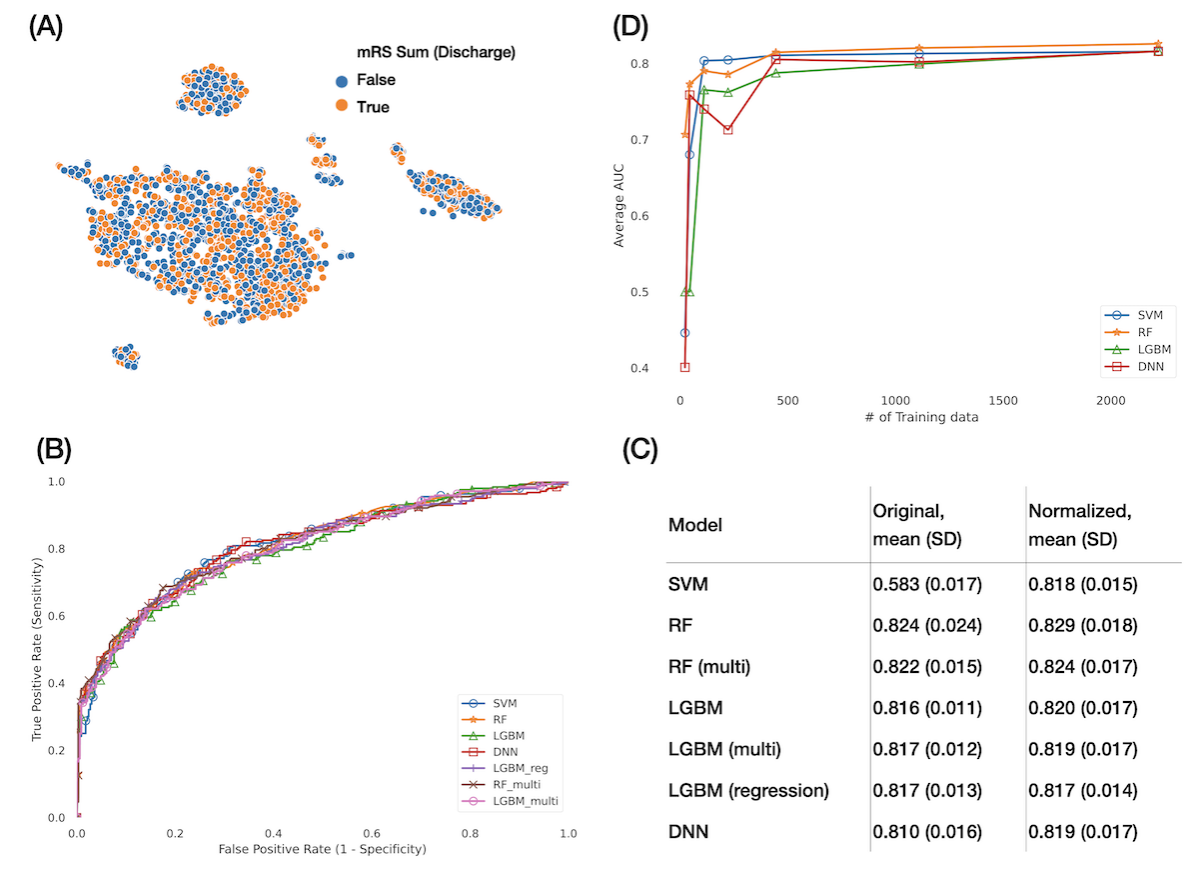
Prediction of modified Rankin Scale (mRS) at hospital discharge. The outcome variable mRS at discharge was transformed from 6 ordinal classes to a binary class. The good outcome was defined by mRS {0,1,2}, whereas the bad outcome was indicated by mRS {3,4,5,6}. (A) The t-SNE graph shows the distribution of the data. Orange indicates discharge mRS 3-6 and blue represents mRS 0-2. (B) ROC curves for 4 machine learning models. (C) Comparisons of AUC between the data with and without normalization of numerical features. (D) AUC for different amounts of data. AUC: area under the curve; DNN: deep neural network; LGBM; light gradient boosting machine; mRS: modified Rankin Scale; RF: random forest; ROC: receiver operating characteristic; SVM: support vector machine; t-SNE: t-distributed stochastic neighbor embedding.

The t-SNE was used for unsupervised clustering to visualize the data. Of the entire data set containing 2780 cases, the 1284 orange dots for a bad outcome and the 1571 blue dots for a good outcome overlapped to a certain degree (Figure 2A). The t-SNE results showed the relationship between the bad and good outcomes at the feature stage, but this does not mean that the machine learning models could not separate the mixed data.

Figure 2B shows the ROC curve for comparing model performances using normalized data. The overlapping curves indicate that the models performed equally well with the AUC being approximately 0.8, with no model being significantly superior to the others. Normalization of the numerical data improved the performance of the SVM model because of its linear nature, but normalization was not beneficial for the tree models and DNN (Figure 2C). We further simulated different volumes of data by sampling different fractions (0*.*01*,* 0*.*02*,* 0*.*05*,* 0*.*1*,* 0*.*2*,* and 0*.*5) of data from the entire training data set, conducted the 5-fold cross-validation, and determined the performance at each data volume (Figure 2D). On increasing the training data to more than 500 samples, the model performance reached a plateau, with the average AUC for RF being near 0.8, almost as high as that for the entire data. With more data, the performance of all the 4 models improved. In contrast, with limited data, the performance would also be acceptable.

**Figure 3:**
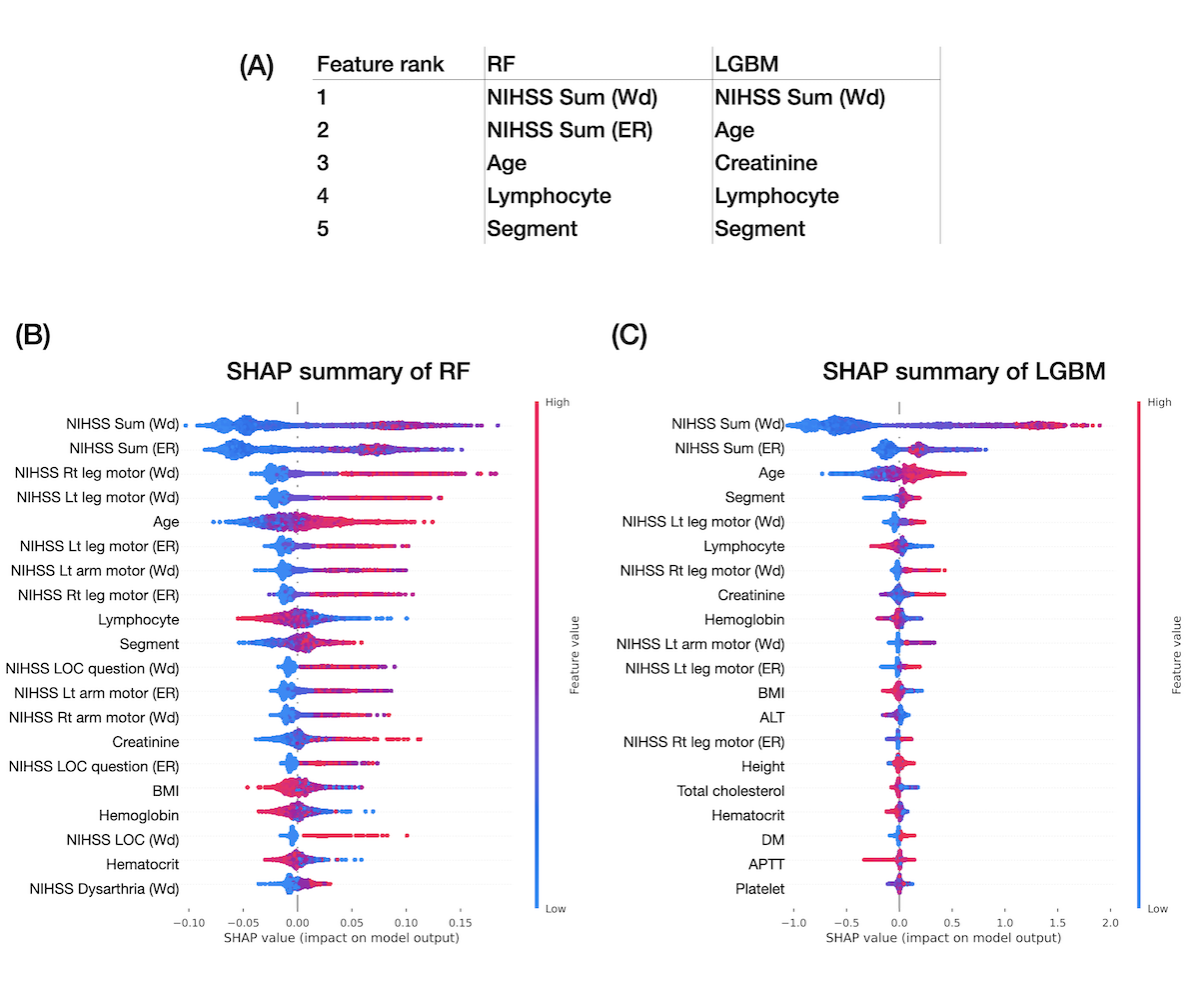
Feature importance for predicting modified Rankin Scale at hospital discharge. (A) Top 5 important features of random forest and light gradient boosting machine. SHapley Additive exPlanations of (B) random forest and (C) light gradient boosting machine. Red indicates higher feature sample values, and blue indicates lower feature sample values. For example, the higher the total National Institutes of Health Stroke Scale scores at emergency room and at ward admission, the more severe would be the stroke outcome. ALT: alanine transaminase; APTT: activated partial thromboplastin time; DM: diabetes mellitus; ER: emergency room; LGBM: light gradient boosting machine; LOC: level of consciousness; NIHSS: National Institutes of Health Stroke Scale; RF: random forest; SHAP: SHapley Additive exPlanations. Wd: ward.

We further applied feature importance and compared it with SHAP in terms of the summary aspect. The top 5 features in RF and LGBM were similar in terms of the NIHSS total score, age, WBC differential counts of lymphocyte and segmented neutrophil, and renal function creatinine (Figure 3A). On the other hand, the SHAP summary of the RF and LGBM models presented the ranking of important features and their influence on predicting outcomes (Figures 3B-3C). For example, the SHAP summary suggested higher NIHSS total scores, worse lower limb motor function, older age, higher segmented neutrophil, and lower lymphocyte percentage of WBC differential counts, indicating a higher mRS score for more dependency at hospital discharge.

### Prediction of In-Hospital Deterioration

Of the initial cohort of 2780 patients, 2622 (94%) were nondeterioration and 158 (6%) were deterioration cases. The coding ratio of in-hospital neurological deterioration, medical problems, brain herniation, and hemorrhagic transformation was 0.64:0.18:0.14:0.04. Next, we compared the performances of the 4 models in predicting deterioration and the 4 resampling methods for imbalanced data. Finally, we compared the feature importance.

**Figure 4:**
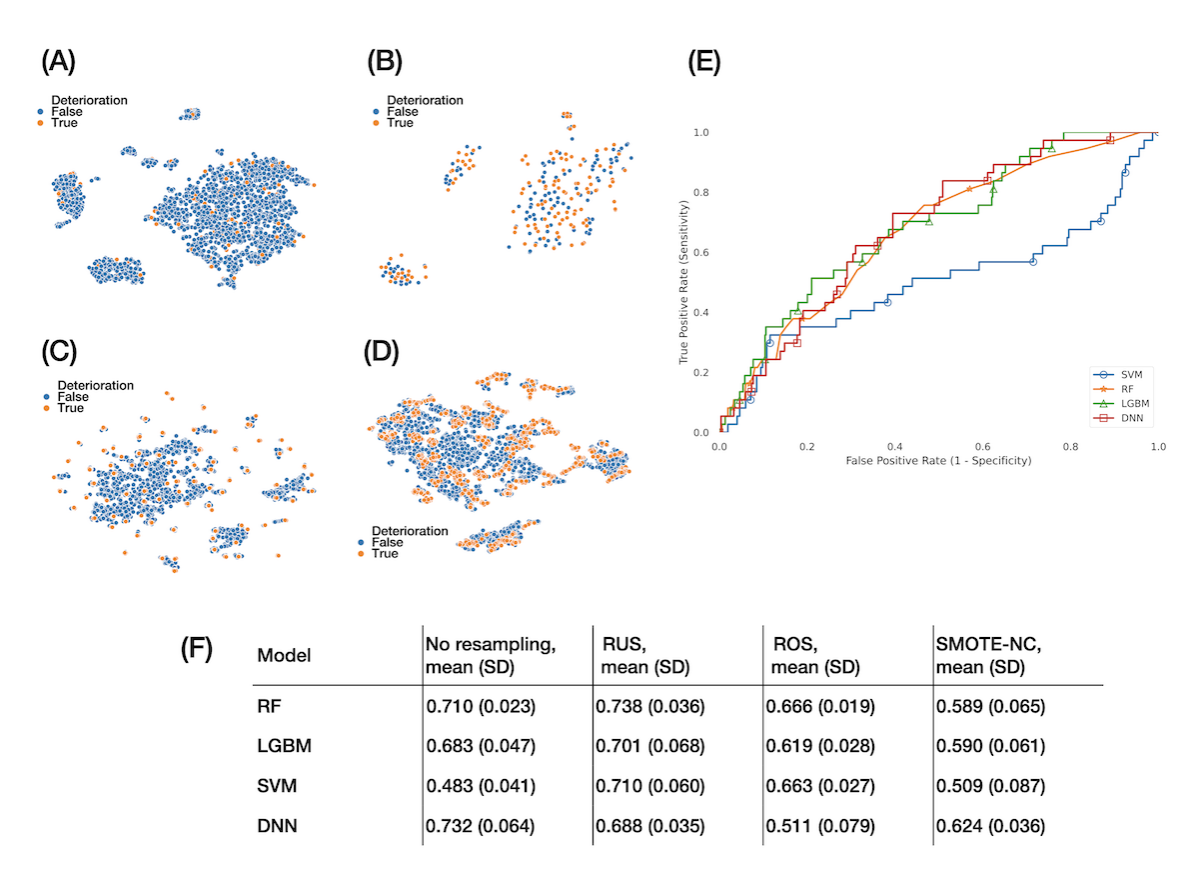
Prediction of in-hospital deterioration. (A) Visualization by t-distributed stochastic neighbor embedding of the original sample shows an imbalanced outcome. The 3 resampling methods processed the imbalanced data with (B) random under sampling decreasing the majority class, (C) random over sampling increasing the minority class, and (D) synthetic minority over-sampling technique with nominal continuous data synthesis from the minority class. (E) Receiver operating characteristic curves for predicting in-hospital deterioration from the data without resampling. (F) Comparison of the area under the curve in the different resampling methods. Random under sampling was a reasonable choice for resampling. It improved the performance of the random forest, light gradient boosting machine, and support vector machine models, but not the deep neural network. The deep neural network performed better on the original data set than on the resampled data set. DNN: deep neural network; LGBM: light gradient boosting machine; RF: random forest; ROC: receiver operating characteristic; ROS: random over sampling; RUS: random under sampling; SMOTE-NC: synthetic minority over-sampling technique-nominal continuous; SVM: support vector machine.

The sample grouped and visualized by t-SNE showed that deteriorations were the minority surrounded by nondeterioration samples (Figure 4A). The resampling methods RUS and ROS did not group samples well (Figures 4B-4C). Finally, the SMOTE-NC produced synthetic data in the neighborhood of true data, but the data were still not grouped well (Figure 4D).

The ROC curves showed the predictive performance for in-hospital deterioration of different models. In the original data set, RF and DNN outperformed SVM and LGBM (Figure 4E, data without resampling). As for each resampling method, RUS improved the performance of all the models except DNN (Figure 4F). The DNN model performed better on the original data set than on resampled data. The performance of SVM was significantly improved by RUS, ROS, and SMOTE-NC.

**Figure 5:**
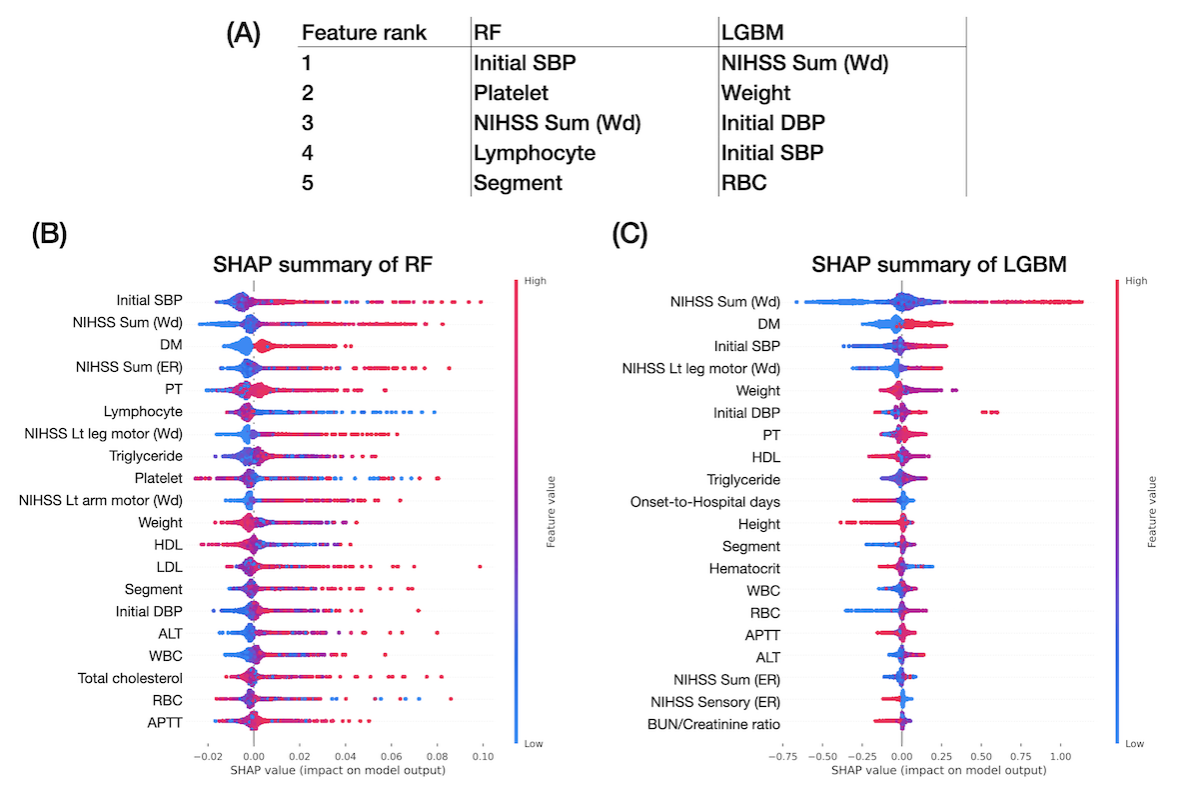
Feature importance for predicting in-hospital deterioration (without resampling). (A) Top 5 important features include initial systolic blood pressure at hospital admission in random forest and light gradient boosting machine. National Institutes of Health Stroke Scale total score at ward admission is also an important feature in both models. SHapley Additive exPlanations of (B) random forest and of (C) light gradient boosting machine. ALT: alanine transaminase; APTT: activated partial thromboplastin time; BUN: blood urea nitrogen; DBP: diastolic blood pressure; DM: diabetes mellitus; ER: emergency room; HDL: high-density lipoprotein; LDL: low-density lipoprotein; NIHSS: National Institutes of Health Stroke Scale; PT: prothrombin time; RBC: red blood cell; SBP: systolic blood pressure; WBC: white blood cell; Wd: ward.

We further compared the top 5 important features with nonresampling data (Figure 5A). The NIHSS total score was critical for predicting in-hospital deterioration. In the SHAP summary, we learned that the higher the NIHSS score, the higher the risk of deterioration. Notably, the initial SBP was prominent in the top 5 important features of RF and LGBM (Figure 5A) and their SHAP summaries (Figures 5B-5C). The SHAP summaries of RF and LGBM showed that the higher the initial SBP, the higher the risk of in-hospital deterioration. In addition, the features obtained from the blood test hemograms, including WBC differential count, platelet count, PT, and RBC, appeared in the top features. Having DM was also crucial in predisposing in-hospital deterioration (Figures 5B-5C).

## Discussion

### Summary

In this study, we used machine learning to predict the mRS outcome at hospital discharge and in-hospital deterioration in the setting of acute ischemic stroke. RF performed the best in most tasks. Applying SHAP to the models combining numerical and higher-dimensional features was feasible, and the SHAP summary emphasized the importance of these features for clinical explanations. As for the resampling of imbalanced data, the effects of resampling on the performance improvement of the models were only equivocal, and SMOTE-NC was not an outstanding method.

Several studies compared models for stroke outcome prediction. In a study with data of over 15,000 patients, DNN outperformed traditional methods when predicting stroke patient mortality [22]. The stroke outcomes predicted by DNN were superior to the ASTRAL scores [23]. However, DNN made no difference in another study predicting 3-month mRS [23]. In our study, DNN did not excel in predicting the discharge mRS, but it performed better than the other models when predicting in-hospital deterioration using nonresampling data. Therefore, DNN is a reasonable choice for the prediction of early deterioration in acute ischemic stroke.

Gradient boosting machine (GBM) and RF are tree-based machine learning models. In a comparative study, extreme gradient boosting (XGBoost) performed better than the traditional GBM in predicting 3-month mRS [24]. However, another study has mentioned that RF performs the best when compared to XGBoost and other traditional models, such as logistic regression, decision tree, and SVM [25]. Similarly, we found that RF performed well in targeting in-hospital deterioration and predicting independence at discharge. RF is effective with imbalanced data and therefore performs well in medical issues with scarce outcomes [26]. RF is suitable for predicting medical diagnosis, and feature ranking helps the RF model in medical classification [27]. Therefore, using RF in predicting early stroke outcomes was feasible. On the contrary, SVM is the least suitable model for stroke early outcome prediction.

### Recent Progress in Model Interpretation

Interpreting how models predict outcomes is sometimes as crucial as their accuracy. In recent years, there has been an increasing amount of literature explaining machine learning models, which helps investigate their learning mechanisms, debug these models, avoid adversarial attacks, and verify the fairness and bias of these models [28,29]. Tree models have some simple inbuilt methods, such as counts for the features used in the model. However, these methods lead to biased approaches, as they tend to inflate the importance of continuous features or high-cardinality categorical variables. To solve the black-box nature of complex models such as deep learning models, the additive feature attribution methods alter the inputs to see how the outputs react and provide a practical solution for the models [30]. The local interpretable model-agnostic explanation, introduced in 2016, approximates a black-box model using a simple linear surrogate model locally [31,32]. Recent explainers, including the SHAP announced in 2018, explore the model from a more global perspective. [21,32]. In a study aiming to predict extubating failure in intensive care units, SHAP analysis proved effective and accurate [33]. With the help of SHAP, we determined the contribution of each feature toward predicting stroke outcomes. The SHAP summary distinguished the features that could separate targets and nontargets from those features that could not.

When working with imbalanced data, SMOTE resampling often achieves better performance in predicting stroke occurrence [34]. However, investigating important features with synthetic data maybe not be persuasive because of its nature of linear interpolation. Repeatedly resampling categorical features could lead to overfitting of the synthetic data. In contrast, continuous features usually stood out without resampling. SMOTE-NC resampling for the imbalanced data of in-hospital deterioration could even worsen model performance. The reason may be the overfitting of categorical data (Figure 4F).

### Initial Blood Pressure in Predicting Early Outcomes of Ischemic Stroke

This work followed the SRICHS registry study, which found the associations between initial blood pressure and 1-year outcomes [35]. In this work, the machine learning models RF and LGBM identified high initial SBP as a crucial factor influencing in-hospital deterioration. High SBP is a strong predictor of stroke [36] and ranks the first among the stroke risk factors contributing to stroke-related DALYs [37]. Chronic hypertension is the most important modifiable risk factor of stroke, according to the INTERSTROKE study [38]. Persistent high blood pressure indicates a worse long-term stroke outcome [39]. High initial blood pressure is detrimental to early neurological outcomes and heralds the deterioration of neurological function in the hospital [40]. Patients with high blood pressure tended to encounter acute infarct volume expansion [41]. Consistent with traditional statistics, our machine learning models supported the importance of blood pressure in predicting early deteriorations in terms of neurological, pathophysiological, and medical changes of acute ischemic stroke. During the creation of this data set, endovascular therapy was not a standard treatment yet. Current studies highlight the importance of blood pressure for stroke patients receiving endovascular therapy [42]. Possessing the capability to process complex data, our machine learning models are promising tools to solve complicated problems in the new era of stroke care, such as blood pressure problems in endovascular therapy.

### DM and Early Stroke Outcomes

DM is a known risk factor for stroke. It accelerates the development of ischemic stroke at a younger age [43]. Compared to nondiabetic stroke patients, ischemic stroke patients with DM had worse neurological deficits, less favorable outcomes from rehabilitation, delayed recovery from the stroke-related deficit, a longer hospital stay for acute ischemic stroke, a higher probability of experiencing a recurrent stroke within 1 year, and a higher rate of 1-year mortality [43,44]. In our study, having DM was a strong predictor for in-hospital deterioration in the SHAP summary of RF and LGBM. Other studies also revealed that DM predisposed early neurological deterioration [45] and increased mortality during hospital stay [43]. This finding suggests that the explainable machine learning model using the SHAP summary is as informative as the stroke registry statistics.

### Limitations of the Study

There were several limitations of this study. First, the registry-based study might have inconsistent assessments and treatments of the patients, incomplete data registration, missing outcomes, and loss of follow-up data [46]. Because of the potentially underreported data, the outcomes might be underestimated. Still, tracking the natural history of a disease, collecting a large number of patients, and yielding generalizable findings make registry-based studies valuable in understanding diseases and outcome assessments. Second, our machine learning models predicted discharge mRS more accurately than in-hospital deterioration. Because general condition deterioration involves multiple factors and individual circumstances, predicting it is more complicated than predicting the neurological status at discharge, which could refer to the initial neurological status. The attributes of the current study design limited the quality and quantity of the features used in model design. In future studies, prospectively collecting delicate parameters, such as continuous vital sign recordings and neuroimages, may improve the performance of these models when predicting in-hospital deterioration. Third, the data set we used in this study was collected in 2009. In the past 10 years, the disease course of ischemic stroke may have changed due to the popularity of comorbidities, demography of stroke proneness, progress in stroke treatment, and improved poststroke care. The machine learning models used in this study may not be completely suitable for new data, and the models may need to be retrained and adjusted. Nevertheless, novel therapies, such as intravenous thrombolysis and endovascular thrombectomy, for acute ischemic stroke were not prevalent a decade ago, and, therefore, we could clearly understand the disease nature course from this data analysis.

### Conclusions

RF, an ensemble algorithm of regression and classification containing multiple decision trees, outperformed SVM, LGBM, and DNN in targeting early stroke outcomes of discharge mRS. RF and DNN performed well in predicting in-hospital deterioration. Using the SHAP summary and feature importance ranking may help clinicians in explaining the prediction of the machine learning models. The multidomain feature bank, combining physiological monitoring values, laboratory data, and neurological severities, as well as the improved performance of the models helped predict in-hospital deterioration. These machine learning models are promising for advanced applications in stroke outcome prediction.

## Abbreviations

ASTRAL: Acute Stroke Registry and Analysis of Lausanne
AUC: area under the curve
DALYs: disability-adjusted life years
DM: diabetes mellitus
DNN: deep neural network
EFB: exclusive feature bundling
ER: emergency room
GOSS: gradient-based one-side sampling
LGBM: light gradient boosting machine
mRS: modified Rankin Scale
NIHSS: National Institutes of Health Stroke Scale
PT: prothrombin time
RBC: red blood cell
RF: random forest
ROC: receiver operating characteristic
ROS: random over sampling
RUS: random under sampling
SBP: systolic blood pressure
SHAP: SHapley Additive exPlanations
SMOTE: synthetic minority over-sampling technique
SMOTE-NC: synthetic minority over-sampling technique-nominal continuous
SRICHS: Stroke Registry of the Chang Gung Healthcare System
SVM: support vector machine
t-SNE: t-distributed stochastic neighbor embedding
WBC: white blood cell
